# Dengue Fever

**Published:** 1851-01

**Authors:** 


### SUMMARY OF RECENT MEDICAL INTELLIGENCE.

---

*Dengue Fever.*—The Charleston Medical Journal, for November, 1850, contains an able memoir on the Epidemic Dengue of Charleston, in the summer of 1850, from the pen of Professor Samuel Henry Dickson. This epidemic differed in some respects from former inflictions of the disease. In 1835, Professor Dickson wrote an admirable monograph on Dengue, and as there are few readers whose medical literature extends that far back, who are not familiar with the general character of the disease, known as Dengue, a character indelibly stamped by Prof. Dickson, we shall confine our synopsis, mainly to the distinctive characteristics of the Charleston epidemic, of 1850, the summer of which year, Dr. Dickson says, "will be remarkable in the medical history of Charleston, for the prevalence of epidemic fever of a singular character. The season had been unprecedentedly hot and dry. The thermometer ranged between 80 and 90° for forty days successively, approaching or even exceeding the highest of these points, and never falling, *within that time*, lower than the lowest." There had been no rain for a long time; "the earth was parched; the sky dazzlingly clear; the nights as well as the days oppressive." There had been a good deal of hooping cough and scarlatina, the latter being severe.

In the latter part of July cases occurred which were considered scarlatina, a mistake not quite equal to some blunders in diagnosis, made a few miles from this city, a few weeks ago, consisting of pronouncing several of the first cases of varioloid, in that locality, attacks of dengue! In the Charleston dengue, "the fever assailed with great violence, ran a brief course, inflicting intense suffering, and disappeared, in many instances, without any cutaneous eruptions." Prickly heat was prevailing at the time, and it is supposed that the dengue eruption may have been overlooked, but Dr. Dickson says, "in the early stage of the epidemic, it was certainly wanting in a large proportion of instances." As the epidemic advanced, "exanthematous contingencies" were more and more certain to exhibit themselves, in the white race. In numerous examples, the blacks were exempt from the eruption.

In a majority of cases, the attacks were sudden and violent. There was a tendency, frequently, to remission and intermission, and the hour of the access of the febrile paroxysm, in a majority of those cases closely observed by Dr. Dickson, was ten in the morning. The course of the disease generally occupied eight days. When the eruption subsided, all ailment usually disappeared slowly and gradually. There was great muscular weakness. In the early part of the epidemic, the eruption was often absent, in the latter part of the summer, it was almost universally present. In the blacks, the eruption was generally absent, in three cases out of five.

The epidemic was very universal. Numerous large households were all attacked. Of Dr. Dickson's family of eleven, he alone escaped. In his kitchen, fourteen out of seventeen were attacked.

Among the visitors to the city, the time of the latent period was various, but sometimes, as Dr. Dickson says, "prodigiously brief." Attacks followed exposure to the air of the city in a very short space of time, usually.

The prognosis was altogether favorable. Violent as the symptoms were in the onset, they subsided after a time, and certain restoration to health followed. Fatal terminations were very rare.

The eruption was various in its character. Ten varieties are named by Prof. Dickson: the scarlatinous, the rubeolus, the érysipelatous, the variolous, or varicellous, the lichenoid, the papulous, the phlegmonoids, the milliary-urticarious, the purpurous, and, in one instance, the lepra vulgaris. It is evident that Dengue is a free trader in "eruptive contingencies."

In Dr. Dickson's former monograph, already noted, he believed that the break bone fever, described by Dr. Rush, as prevalent in Philadelphia in 1780, was a different disease from the Charleston epidemic of 1828. He now believes them identical diseases, except in some modifications.

Dr. Dickson discusses the exanthenatous character of dengue, very ably, and cautiously concludes, that "it may occupy a middle position between maculated typhus, and the exanthemata proper, such as small pox, measles, and scarlutina."

Of the cause of dengue, Dr. Dickson says but little, and we regret that that little is not to the point. Strangely enough, he looks upon dengue as contagious. In the gloom and obscurity of the causes of disease, occasional occurrences loom up that are mistranslated into contagion, and they are thus mistranslated generally on the same principle that Hahneman ascribed seven-eighths of chronic diseases to the *psoric virus*—he had to say something, and it was as easy to say that as anything else. Contagion, as a cause of disease, is like "sympathy" once was, as an explanation of pathological phenomena—an easy stool to lounge upon. We have not a doubt that during the prevalence of the prickly heat, at Cnarleston, of which Dr. Dickson speaks, as many facts, to prove it contagious, were developed, as there were in dengue. The analogies, on whose bewildering, and uncertain seas, Dr.

Dickson launches his bark, do not bear him up. Dengue is evidently a disease of hot climates, and unquestionably of hot weather. Can Dr. Dickson inform us of any contagious exanthematous disease that respects the geography of the zones? Even the vegetable analogies to which he appeals—the nut-grass, the cockle-bur and the dandelion desert the Professor on this point. If they move in the current of migratory bodies of men, they can also grow wherever the sun and earth can call vegetable life into growth. Does, or can dengue follow this law? If so, where are the instances?

Dr. Dickson remarked an unusual prevalence of boils, and carbuncles during the late epidemic. We shall have more to say on this subject in a paper on Variola, in a future number of this Journal.

From Dr. Dickson's account of the treatment of den gue, it is evident that the disease would have afforded abundant glorification for the Homœopathists. But little treatment of any kind was needed. B.

